# Clinical application of non-invasive prenatal diagnosis of phenylketonuria based on haplotypes via paired-end molecular tags and weighting algorithm

**DOI:** 10.1186/s12920-021-01141-4

**Published:** 2021-12-17

**Authors:** Dai Peng, Zhao Ganye, Sun Gege, Xia Yanjie, Liu Ning, Kong Xiangdong

**Affiliations:** The Genetics and Prenatal Diagnosis Center, The Department of Obstetrics and Gynecology, The First Affiliated Hospital of Zhengzhou University, Zhengzhou, 450052 Henan China

**Keywords:** Phenylketonuria, Non-invasive prenatal diagnosis, Haplotype, Weighting algorithm, Paired-end molecular tags

## Abstract

**Background:**

Phenylketonuria (PKU) is a metabolic disease that can cause severe and irreversible brain damage without treatment.

**Methods:**

Here we developed a non-invasive prenatal diagnosis (NIPD) technique based on haplotypes via paired-end molecular tags and weighting algorithm and applied it to the NIPD of PKU to evaluate its accuracy and feasibility in the early pregnancy. A custom-designed hybridization probes containing regions in phenylalanine hydroxylase (*PAH*) gene and its 1 Mb flanking region were used for target sequencing on genomic and maternal plasma DNA (7–13 weeks of gestation) to construct the parental haplotypes and the proband’s haplotype. Fetal haplotype was then inferred combined with the parental haplotypes and the proband’s haplotype. The presence of haplotypes linked to both the maternal and paternal mutant alleles indicated affected fetuses. The fetal genotypes were further validated by invasive prenatal diagnosis in a blinded fashion.

**Results:**

This technique has been successfully applied in twenty-one cases. Six fetuses were diagnosed as patients carrying both of the mutated haplotypes inherited from their parents. Eleven fetuses were carriers of one heterozygous *PAH* variants, six of which were paternal and five of which were maternal. Four fetuses were absence of pathogenic alleles. All results were consistent with the prenatal diagnosis through amniotic fluid.

**Conclusions:**

The results showed that our new technique applied to the genotyping of fetuses with high risk for PKU achieves an accurate detection at an early stage of pregnancy with low fetal fraction in cell free DNA.

**Supplementary Information:**

The online version contains supplementary material available at 10.1186/s12920-021-01141-4.

## Background

Phenylketonuria (PKU) is an autosomal recessive inherited disease, which is mainly caused by a mutation in the phenylalanine hydroxylase (*PAH*) gene, including 60% is missense mutations, and other common variants being splice variants and deletions. As a result, this mutation triggers the decrease or deficiency in the PAH activity, elevation of phenylalanine concentration in the blood and accumulation of phenylalanine in the blood and nervous system [1, 2]. In China, the overall incidence rate is about 1/16,000 [3], showing regional differences, such that the incidence rate in northern China is higher than that in southern China [4]. The typical manifestations of PKU include mental retardation, light hair and skin color, eczema, epilepsy, hyperactivity, a musty smell of sweat and urine. Through early screening, diagnosis and treatment, the damage in the nervous system can be avoided and the level of intellectual and physical development can be ensured in children with PKU [5, 6]. It is obvious that prenatal diagnosis is the major method to prevent PKU in newborns, specifically in families that already have PKU child patients. Currently, the frequently used methods for prenatal diagnosis include chorionic villus sampling and amniocentesis [7]. However, these procedures may lead to maternal bleeding, abortion or intrauterine infection. Besides, some pregnant women have contraindications for prenatal diagnosis, such as in the case of threatened abortion, preoperative body temperature > 37.2 ℃, bleeding tendency or intraluminal infection. In 1997, Lo’s team discovered fetal cell-free deoxyribonucleic acids (cf-DNAs) in the peripheral blood of pregnant women. This discovery opened the door for a new non-invasive prenatal test (NIPT) for diseases such as fetal chromosome aneuploidy, chromosome microdeletion/duplication syndromes and monogenic inherited diseases [8–11], offering a new option for couples concerned about the risk of prenatal diagnosis. Although the use of NIPT for fetal chromosome aneuploidy, microdeletion/duplication syndromes and single gene disorders has already been put into clinical application abroad [12], but it has not been used in clinical practice in China. In this study, we successfully applied non-invasive prenatal diagnosis (NIPD) for PKU in clinic and evaluated its feasibility of this clinical application. Out work provides a program for the earlier and safer detection of PKU in clinical high-risk families.

## Methods

### Subjects

The PKU high-risk families treated in the Genetics and Prenatal Diagnosis Center of the First Affiliated Hospital of Zhengzhou University from September 2020 to May 2021 were enrolled in this study. In each of these families, both the pregnant woman and partner were carriers of the *PAH* gene mutation. Hence, 25% of their fetuses were PKU patients, 50% were carriers and 25% were normal. The whole program consisted of two stages. The first stage is method establishment. The NIPD technique for PKU was established using the peripheral plasma of pregnant women. The plasma was prepared during fetal chorionic villus sampling and gene diagnosis at weeks 11-13 of pregnancy [average: (11.6+2.0) weeks, n = 9]. The second stage is method application. The established NIPD technique for PKU was clinically applied to detect variations or mutations using peripheral blood of pregnant women. The plasma was collected at < 11 weeks of pregnancy [average: (8.0+2.1) weeks, n = 12] from pregnant women who were informed of the prenatal diagnosis, and the feasibility of the method was verified. All the pregnant women signed informed consents and were willing to undergo invasive prenatal diagnosis, such as chorionic villus sampling or amniocentesis.

### Design and synthesis of the probe

According to the experimental design, the required capture efficiency of the *PAH* gene should be 20-50%, and the site coverage with sequencing depth of 20 X should be > 98%. Therefore, we selected the *PAH* gene and its upstream and downstream flanking regions (1 Mb each). Based on the gnomAD database, single nucleotide polymorphism (SNP) loci were screened according to the carrying ratio of > 10% loci (10-90% mutation frequency) in East Asian population, and 3,849 SNP loci were obtained. Besides, the ribonucleic acid (RNA) probe was synthesized using iGeneTech.

### Capturing and sequencing of the target sequence

Firstly, 3 mL of peripheral blood was separately collected from the probands and fathers, and 10 mL of peripheral blood was drawn from the pregnant women. Then, genomic DNA was extracted from all the peripheral blood samples using the whole blood genomic nucleic acid extraction reagent (606001, Zeesan Biotech, China). Next, 4,000 ng of whole genomic DNA was broken into target fragments with the length of 150-200 bp using a Bioruptor Pico sonication system (Diagenode, Belgium), and the size of the nucleic acid fragments was detected using electrophoresis. Afterwards, cf-DNA was extracted from 3 mL of the pregnant women plasma using the free nucleic acid extraction kit (55114, Qiagen, Germany) and following the manufacturer’s instructions. After end repair and A-tailing of the target fragment and cf-DNA (K8504, KAPA Hyper Prep Kit, America), the xGen Duplex Seq adapters (10006914, IDT, America) were linked to both ends of the target DNA, and polymerase chain reaction (PCR) amplification and enrichment of the library with tagged primers were accomplished.

After that, 400 ng of the peripheral blood DNA library and 600 ng of the cf-DNA library were mixed, dried with the Concentrator plus vacuum concentrator (Eppendorf, Germany) and dissolved with DEPC water. Then, hybridization buffer, probe and blocking sequence were successively added and kept at 95 ℃ for 5 min. Later, it was covered with a hot cover, hybridized for ≥ 20 h at 65 ℃ and purified. Then, another round of PCR amplification and purification was performed, and the library for detection was obtained. The library was quantified (Qubit 2.0, America), and fragment analysis (Qsep100, Taiwan, China) was performed. Finally, the original sequencing data were obtained by subjecting the library to PE150 sequencing using NovaSeq 6000 (Illumina, America).

### Determination of fetal genotype

#### Sequencing data filtering and SNP calling

Through error correction by introducing paired-end molecular tags [13] and bioinformatics software, the background noise of the PCR amplification and sequencing errors was reduced, and the low frequency variation was accurately detected (< 1%). The Fastq file was converted into unmapped BAM (uBAM) format using the *FastqToSam* (Picard, 2.19.2) tool, the sequence paired-end molecular tag information was extracted using the *ExtractUmisFromBam* (Fgbio, 0.8.0) tool and stored in the RX tag of the uBAM file to be analyzed later, and then the uBAM file was converted into a BAM file using the *MergeBamAlignment* (Picard) tool. The *GroupReadsByUmi* (Fgbio) tool was used to identify the reads of the same molecular origin according to the information of the start and end positions of the sequence and paired-end molecule tag in the BAM file, and the parameter min-map-q = 20, edits = 1 was used to filter out low quality sequences. The filtering conditions (error-rate-pre-umi = 45, error-rate-post-umi = 30) were set using the *CallDuplesConsensusReads* (Fgbio) tool to obtain the consensus sequences required by multiple analyses. Next, the sequences were filtered according to the tag value generated by the reads’ analysis of the consensus sequences using the *Filterconsensusreads* (Fgbio) tools as follows. (A) Consensus sequences with support values lower than min-reads were filtered out, including paired-end consensus sequences with min-reads = 2, consensus sequences with min-reads = 1 of one single chain and consensus sequences with min-reads = 1 of the other single chain. (B) Consensus sequences with an error rate higher than max-reads-rate = 0.05 were filtered out. (C) Bases with a base error rate higher than max-rate-error-rate = 0.1 or a quality value lower than min-base-quality = 50 were obscured and marked with N. (D) Sequences with N base ratio higher than max-no-call-fraction = 0.05 were filtered out. Overlapping regions were clipped and removed using the *ClipBam* (Fgbio) tool to avoid repeated counting of the same mutation. Finally, the *VarDict* (1.5.1) tool was used for mutation analysis, and the results were generated as a VCF file.

### Calculation of fetal fraction in cell free DNA

In the target region, the SNP loci with homozygotes that are not of the same type in the parents were selected. For example, the maternal genotype was assumed to be AA and the parental genotype was assumed to be TT, then the fetal genotype was AT. The letter p represents the number of reads obtained by paternal specific allele detection, q means the number of reads obtained by allele detection with the same fetal and maternal genome, so the sequence number of A was q and that of T was p, and f represents the concentration of free fetal DNA in maternal plasma, calculated as f = 2×p/ (p+q) [14]. According to the results of fetal DNA concentration, the free fetal fraction in cell free DNA was calculated as follows:

$${\text{e}} = \frac{{\sum\limits_{i = 1}^{n} {fi} }}{n}$$

where n denotes the total calculation site data of the free fetal fraction in cell free DNA.

### Determination of fetal genotype

Based on Mendel’s law of inheritance and using the genetic information obtained through capturing and sequencing of the family (fetus and both parents), the haplotypes of the parents and their linkage with *PAH* were constructed, and the fetal genotype came from the genetic spectrum of the parents. Next, according to the family genotype combination, the loci were divided into 8 groups (Table 1), and the genotypic pathogenicity ratio and wild type allele ratio of each group were calculated. The genotypic ratios of the qualified loci in each group were combined, and the average number of all loci in each group was calculated. Then, the haploid risk value (S_value) of a single type was calculated. We defined 0 as the haplotype related to the pathogenic mutation, and 1 as the haplotype related to wild type allele. Paternal inheritance was determined by SNPs that are heterozygous in the fathers but homozygous in the mothers, while maternal inheritance was determined by SNPs that are heterozygous in the mothers but homozygous in the fathers. S1-S4 were determined as maternal haplotypes and S5-S8 as paternal haplotypes [15]. In addition, a weighting algorithm model was constructed to determine the genetic mode of the parental haplotypes by the plasma data. Through this weighting algorithm, the values of the maternal and the paternal haplotypes were calculated, i.e., the maternal haplotype value S_mother and the paternal haplotype value S_father (using the following formulas):

$${\text{S}}\_mother = \prod\limits_{i = 1}^{n = 4} {\left( {\frac{(FEi - BEi)}{{(FEi - PEi)}}} \right)}$$

$${\text{S}}\_{\text{father}} = \coprod\limits_{i = 5}^{n = 4} {\left( {\frac{(FEi - BEi)}{{(FEi - PEi)}}} \right)}$$

where BE and PE denote the ratio of mutant genotypes when fetuses carry wild type allele and mutant haplotypes based on the fetal fraction ratio in cell free DNA, respectively. The specific algorithm is shown in Table 1. FE and ME represent the average value of fetal mutation ratio and the average mutation ratio of maternal loci in the corresponding combination of 8 genotypes, respectively, as calculated by the following formulas:

$${\text{FE}} = \frac{{\sum\limits_{i = 1}^{n} {Ri} }}{n}$$

$$ME = \frac{{\sum\limits_{i = 1}^{n} {{\text{MR}}i} }}{n}$$

where MR represents the mutation ratio of a single locus, and the mutation ratio R = AD/DP, where AD is the number of the mutant type reads, and DP is the number of the wild type + mutant type reads.

**Table 1:** Parent haplotype genotype group

Type	Paternal genotype	Maternal genotype	Proband genotype	The proportion of pathogenic haploid mutations (PE)	The proportion of benign haploid mutations (BE)	Note
S1	0/0	0/1	0/0	ME-e/2	ME	Determination of maternal haploid genotype
S2	0/0	0/1	0/1	ME	ME-e/2	
S3	1/1	0/1	0/1	ME	ME+e/2	
S4	1/1	0/1	1/1	ME+e/2	ME	
S5	0/1	0/0	0/0	0	e/2	Determination of paternal haploid genotype
S6	0/1	0/0	0/1	e/2	0	
S7	0/1	1/1	0/1	1-e/2	1	
S8	0/1	1/1	1/1	1	1-e/2	

According to the results of S_values, fetal genotypes were inferred as follows: if S_values of the maternal and paternal haplotypes were ≥ 10, fetal genotypes would then be pathogenic haplotypes; if they were ≤ 0.1, fetal genotypes would be wild type allele haplotypes; if they were > 0.1 and < 10, and the pathogenicity of the fetal genotypes would then be undeterminable.

### Invasive prenatal genetic detection

NIPD is similar to NIPT, also an indirect diagnostic technique. The genotype of the fetus is determined based on the results of the sequencing of free DNA in the peripheral blood of the pregnant woman. There may be discrepancies between the test results and the genotype of the fetus, because the main source of cf-DNA is the cells of trophoblastic origin released from the syncytiotrophoblast in the form of syncytial knots, and not the fetal unit itself [16], so invasive prenatal diagnosis of the fetus is necessary. After iodophor disinfects the skin at the puncture site, parental diagnosis was verified through ultrasound-guided chorionic villus sampling performed at 11-13 weeks of pregnancy or amniocentesis performed at 18-24 weeks of pregnancy. According to the gene detection results of both parents and proband previously (Table 2), we also verified its genotype (missense mutations or deletions) by sanger sequencing or multiplex ligation-dependent probe amplification (MLPA), then DNA was extracted from the chorionic villi or amniotic fluid, and the fetal genotype was detected using Sanger sequencing or MLPA to determine the accuracy of NIPD.

**Table 2:** Results of 21 PKU families by NIPD and invasive diagnosis

Pedigree	Proband	Father	Mother	NIPT results	Invasive test results	Consistency
F01	c.782G>A(p.Arg261Gln) c.194T>C(p.Ile65Thr)	c.194T>C(p.Ile65Thr)	c.782G>A(p.Arg261Gln)	Affected	Affected	Y
F02	c.782G>A(p.Arg261Gln) c.1068C>A(p.Tyr356Ter)	c.782G>A(p.Arg261Gln)	c.1068C>A(p.Tyr356Ter)	Affected	Affected	Y
F03	c.526C>T(p.Arg176Ter) c.1197A>T(p.Val399Val)	c.1197A>T(p.Val399Val)	c.526C>T(p.Arg176Ter)	Paternal mutation carriers	Paternal mutation carriers	Y
F04	c.721C>T(p.Arg241Cys) c.728G>A(p.Arg243Gln)	c.728G>A(p.Arg243Gln)	c.721C>T(p.Arg241Cys)	N	N	Y
F05	c.442-1G>A(p.IVS4-1G>A) c.1197A>T(p.Val399Val)	c.1197A>T(p.Val399Val)	c.442-1G>A(p.IVS4-1G>A)	Paternal mutation carriers	Paternal mutation carriers	Y
F06	c.728G>A(p.Arg243Gln) c.728G>A(p.Arg243Gln)	c.728G>A(p.Arg243Gln)	c.728G>A(p.Arg243Gln)	Paternal mutation carriers	Paternal mutation carriers	Y
F07	c.331C>T(p.Arg111Ter) c.611A>G(p.Tyr204Cys)	c.611A>G(p.Tyr204Cys)	c.331C>T(p.Arg111Ter)	Affected	Affected	Y
F08	c.1194A>G(p.Lys398Lys) c.1238G>C(p.Arg413Pro)	c.1238G>C(p.Arg413Pro)	c.1194A>G(p.Lys398Lys)	Affected	Affected	Y
F09	c.611A>G(p.Tyr204Cys) c.728G>A(p.Arg243Gln)	c.611A>G(p.Tyr204Cys)	c.728G>A(p.Arg243Gln)	Paternal mutation carriers	Paternal mutation carriers	Y
F10	c.611A>G(p.Tyr204Cys) c.782G>A(p.Arg261Gln)	c.611A>G(p.Tyr204Cys)	c.782G>A(p.Arg261Gln)	N	N	Y
F11	c.721C>T(p.Arg241Cys) c.721C>T(p.Arg241Cys)	c.721C>T(p.Arg241Cys)	c.721C>T(p.Arg241Cys)	Paternal mutation carriers	Paternal mutation carriers	Y
F12	c.721C>T(p.Arg241Cys) c.331C>T(p.Arg111Ter)	c.721C>T(p.Arg241Cys)	c.331C>T(p.Arg111Ter)	Maternal mutation carriers	Maternal mutation carriers	Y
F13	c.968C>T(p.Thr241Ile) c.208_210delTCT(p.Ser70del)	c.208_210delTCT(p.Ser70del)	c.968C>T(p.Thr241Ile)	Paternal mutation carriers	Paternal mutation carriers	Y
F14	c.232_235delGAAT(p.Glu78Phefs*13) E5_E6del	c.232_235delGAAT(p.Glu78Phefs*13)	E5_E6del	Maternal mutation carriers	Maternal mutation carriers	Y
F15	c.116_118delTTC(p.Phe39del) c.526C>T(p.Arg176Ter)	c.116_118delTTC(p.Phe39del)	c.526C>T(p.Arg176Ter)	Affected	Affected	Y
F16	c.482T>C(p.Phe161Ser) c.1197A>T(p.Val399Val)	c.482T>C(p.Phe161Ser)	c.1197A>T(p.Val399Val)	N	N	Y
F17	c.722delG(p.Arg241Profs) c.1238G>C(p.Arg413Pro)	c.1238G>C(p.Arg413Pro)	c.722delG(p.Arg241Profs)	Maternal mutation carriers	Maternal mutation carriers	Y
F18	c.728G>A(p.Arg243Gln) c.442-1G>A(IVS4-1G>A)	c.728G>A(p.Arg243Gln)	c.442-1G>A(IVS4-1G>A)	Maternal mutation carriers	Maternal mutation carriers	Y
F19	c.331C>T(p.Arg111Ter) c.1301C>A(p.Ala434Asp)	c.1301C>A(p.Ala434Asp)	c.331C>T(p.Arg111Ter)	N	N	Y
F20	c.728G>A(p.Arg243Gln) c.728G>A(p.Arg243Gln)	c.728G>A(p.Arg243Gln)	c.728G>A(p.Arg243Gln)	Affected	Affected	Y
F21	c.442-1G>A(p.IVS4-1G>A) c.875C>T(p.Pro3292Leu)	c.875C>T(p.Pro3292Leu)	c.442-1G>A(p.IVS4-1G>A)	Maternal mutation carriers	Maternal mutation carriers	Y

c, a mutation in the nucleic acid level; p, mutations in protein levels, Y, consistency represents comparison the NIPT results with invasive testing results; N, normal.

## Results

### Output of high-throughput sequencing data

In this study, the following requirements applied: the sequencing data were from the probands and parents’ genomic DNA sequencing with a depth > 500 X, the data amount was > 1.0 G, and the depth of maternal fetal cf-DNA sequencing was > 1,000 X with a data amount of > 2 G. Therefore, the amount of data for one family was equivalent to the whole exome sequencing data of one person. The average coverage of the 20 X target region of genomic DNA and plasma cf-DNA of the 21 families was 99.84% (99.19% - 100%), the average amount of sequencing data was 3.17 G (1.02 G -9.30 G), and the average capture efficiency in the target region was 39.01% (20.11 %-49.56%). After removing the low-quality readings and readings with repeated PCR and multiple calibrations from the original sequencing data, the updated average sequencing depth of genomic DNA in all the families was 1,889.47 X, and the updated average sequencing depth of plasma cf-DNA was 3,020.59 X. In addition, the average duplication rate of genomic DNA was 0.26% (0.07%-0.50%), and the average sequencing duplication rate of plasma samples was 0.46% (0.18%-0.64%). The results are shown in Additional file 1: Table S1.

### Fetal fraction of cell free DNA in plasma

The SNP loci with a heterozygote in the father and a homozygote in the mother or homozygotes of different types in both parents were selected, and based on the information of the SNP loci provided by plasma sequencing and used the formula ${\text{e}} = \frac{{\sum\limits_{i = 1}^{n} {fi} }}{n}$ to calculate the fetal fraction in cell free DNA.

The fetal fraction of cell free DNA in plasma in these 21 families was 3.6-9.7% (average: 6.4%, SD: 1.68%), as shown in Table 3, which indicated intraindividual differences.

**Table 3:** The gestational week and fetal DNA fraction of 21 pregnant women

Pedigree	Gestational week	Fetal DNA fraction (%)
F01	11+1	3.8
F02	10+2	8.1
F03	13+0	5.0
F04	11+1	7.7
F05	13+2	9.7
F06	12+3	8.7
F07	12+6	5.8
F08	11+1	5.8
F09	11+2	5.6
F10	8+1	6.8
F11	8+3	6.7
F12	8+6	4.7
F13	9+4	5.4
F14	7+0	5.1
F15	8+0	6.3
F16	8+2	7.9
F17	9+0	9.0
F18	8+0	5.1
F19	7+2	6.3
F20	7+5	3.6
F21	9+2	7.6

### Results of the prenatal tests of the fetus

In light of Mendel’s law of inheritance, we successfully constructed the parental haplotypes and linked pathogenic mutations based on the SNP information of sequences in the *PAH* gene and its upstream and downstream flanking regions (1 Mb each) in the probands and parents. In the target region, 100-1,284 SNPs were identified per sample. The average number of SNP loci used to predict maternal and paternal haplotypes of the fetus was 412 (ranged: 34-877) and 299 (range: 28-638), respectively. According to the genotype combination listed in Table 1, the paternal or maternal haplotypes of the fetuses were determined using plasma data based on the weighting algorithm. For example, in Family 2 (F2) and F11, the number of allele SNPs supporting paternal haplotypes in the fetus was 548 and 281, while the number of those supporting maternal haplotypes was 677 and 655, respectively (Additional file 1: Table S2). As per the weighting algorithm, the paternal S_value of F2 was 3,280, and the maternal S_value of F2 was 447; since both S_values were > 10, the fetus inherited the paternal and maternal pathogenic haplotypes and suffered from PKU (Additional file 1: Table S2). However, the paternal S_value of F11 was 647, and the maternal S_value of F11 was 0.02. Since the maternal S_value was < 0.1, the fetus inherited maternal wild type allele haplotypes and was a PKU carrier (Additional file 1: Table S2). Based on this strategy, we retrospectively detected the *PAH* genotypes of fetuses in F1-F9. The results showed that 1 fetus was absence of pathogenic alleles, 4 fetuses carried paternal pathogenic haplotypes, and 4 fetuses had PKU, which was consistent with the findings of the invasive genetic diagnosis, as listed in Table 2. A total of 12 fetuses with a high risk for PKU underwent clinical examination at < 11 weeks of pregnancy in F10-F21. The results showed that 3 fetuses were normal, 2 fetuses carried paternal pathogenic haplotypes, 5 fetuses carried maternal pathogenic haplotypes, and 2 fetuses suffered from PKU, which was in accordance with the results of the invasive gene diagnosis at a later stage (Table 2). The results of the retrospective testing and clinical application proved that both the accuracy and specificity of this method were 100%, with no false negative cases.

## Discussion

The use of NIPD in monogenic inherited diseases has become a new trend in clinical practice ever since the discovery of maternal plasma cf-DNA. A previous study reported that the fetal genome map constructed by massively parallel sequencing of maternal plasma cf-DNA can offer a theoretical basis to achieve non-invasive prenatal diagnosis of many genetic diseases and chromosomal alterations [14]. In this work, we designed the RNA probe of the *PAH* gene based on the genetic characteristics of PKU, captured and sequenced the target region of genomic DNA in the peripheral blood and plasma of the parents and probands and obtained the wild type allele and pathogenic haplotypes of each pregnant woman and her partner. Then, each SNP in maternal plasma DNA data was analyzed and counted, and fetal genotypes were inferred based on the established weighting algorithm. The results of the retrospective study and clinical application were consistent with those of invasive gene diagnosis, which proves that the method used in this study is feasible for the early detection of fetuses with a high risk of PKU.

Researchers have studied the use of NIPD for the detection of PKU. Duan *et al.* used circulating single-molecule amplification and resequencing technology (cSMART) to perform retrospective NIPD in 18 PKU families at 11-23 weeks of pregnancy for the first time and reported similar results to those of invasive prenatal diagnosis [17]. In detail, a set of reverse primers covering the *PAH* gene mutation known in the 18 PKU families was designed to perform the cSMART analysis. The results revealed that with a fetal fraction in cell free DNA of > 5%, the mutation ratio values of the maternal mutation site of <47.5%, >47.5% and < 52.5%, and >52.5% indicate no mutation, heterozygous mutation, and homozygous mutation at the site of the fetus, respectively. Although this technique facilitates the diagnosis and confirms the presence of the pathogenic variation, it suffers from some disadvantages. On the one hand, it requires clarifying the parental mutation site for the design of targeted primers. On the other hand, determining the fetal genotype depends on the plasma DNA mutation ratio [17]. The work of Lv *et al.* also presented the use of cSMART to blindly analyze 33 pregnant women with a high risk for PKU at 16-21 weeks of pregnancy. The results showed that cSMART realized the accurate genotyping of 32 fetuses, with an accuracy of 96.7%, and the sensitivity and specificity were 100% and 96.15%, respectively, with no false-negative cases. Besides, the average fetal fraction in cell free DNA was (10.7±2.4) % (range: 7.0-17.3%) [18]. In the aforementioned study, a set of high-density targeted primers (95% coverage) that cover the coding region of the entire *PAH* gene and the intron region of the proximal exon were designed to be used in the sequencing. Through statistical weighting of the mutation frequency, fetal concentration and total number of allele sequences, the maximum likelihood algorithm was introduced to determine the fetal genes. However, this method had the drawback of failing to analyze the pathogenic mutations that might be hidden in the intron region of the distal exon and the proximal 5’- end and distal 3’- end non-translational regions. As a result, it is not applicable for couples with rare mutations in non-exon regions of the *PAH* gene [18]. In another study, Ye and colleagues successfully identified the genotypes of 13 fetuses of 16-20 weeks pregnant women in PKU families using the haplotype-assisted NIPD based on hidden Markov model. The reported results were in agreement with those obtained by the invasive method, achieving an accuracy of 100%, with an average fetal fraction in cell free DNA was (8.86±2.4)% (range: 4.52-20.43%). However, this method also had the limitation that it might be influenced by the determination of gene recombination interference haplotypes and the distinction between the proband and fetal gene recombination [19].

In our study, the haplotype principle was combined with a weighting algorithm to conduct NIPD in 21 fetuses with a high risk for PKU, and the results were consistent with those of invasive prenatal diagnosis. Unlike the reported non-invasive detection of PKU, the technique in our study showed the following advantages: (1) it realized the detection of fetuses with a high risk for PKU at the early stage of pregnancy, without knowing the specific mutation sites of the proband, parents, and the results were in line with those obtained thought prenatal diagnosis. The gestational week of pregnant women was more than week 11 in the previous study, while the gestational week of the pregnant women was less than week 11 in the second stage of our study, and the gestational week of one pregnant woman was week 7, which realized NIPD at the early stage of pregnancy. Hence, if the fetus is determined to have PKU in the early stage of pregnancy, the couples has the right to make informed reproductive choice. Early diagnosis can ensure early intervention through diet and professional healthcare, which will significantly reduce the risk of severe brain damage. (2) The use of single-molecular tags combined with bioinformatics methods allowed for accurate analysis of the fetal low-ratio mutation and the detection at low fetal fraction in cell free DNA, which reduced the influence of experimental contamination and other errors on the experimental results. Previous studies have suggested that during NIPD of fetal chromosome aneuploidy, an excessively low fetal fraction in cell free DNA would result in false-negative results; thus, a fetal fraction in cell free DNA of ≥ 4% is considered a vital quality control index [20, 21]. In non-invasive detection of monogenic diseases, fetal fraction in cell free DNA represents a major parameter in the analysis of site variation. Hence, accurate estimation of fetal fraction in cell free DNA is particularly important. However, many factors like the pregnant women’s gestational age, body weight and others have a great influence on the fetal fraction in cell free DNA [22]. According to the recommendation of the American College of Medical Genetics and Genomics, a fetal fraction in cell free DNA of fetal aneuploidy of < 4% is not suitable for fetal non-invasive detection. In our study, the NIPD technique has made progress in the detection of low fetal fraction in cell free DNA (less than 4%), and the results were in line with those obtained through prenatal diagnosis. However, there are 2 cases of fetal fraction in cell free DNA in the 21 samples were less than 4%, so more samples with low fetal fraction in cell free DNA are needed to verify the feasibility of this technique. (3) Instead of the predicted average value (0.5) of all loci of the possible types, their actual ratio was adopted as the theoretical maternal heterozygous mutation ratio. For example, the average value 0.5 is used to calculate the upper and lower boundaries of the sequential probability ratio test (SPRT) curve when the relative haplotype dosage (RHDO) analysis combined with SPRT is used for haplotype construction [14, 23]. (4) Instead of using the traditional hidden Markov model for the fetus pathogenic haplotypes analysis, the strategy of genotype classification was adopted to determine the average value of multiple sites in a single genotype combination based on the possibility analysis scheme of a single site; this served as the basis for the determination of the pathogenic haplotypes of this genotype combination. Meanwhile, to comprehensively determine the source of parental or maternal haplotypes, 4 groups of parental or maternal genotype combinations were taken as 4 groups of independent events to ensure highly reliable results. When evaluated in clinical practice, the multi-sample detection results demonstrated the good data stability and robustness of our algorithm.

In the present study, measures such as the design optimization of the capture probe, mixed capture of multiple samples and combination of different sequencing technologies were adopted to reduce the NIPD cost. Specifically, to design the RNA probe, the highly heterozygous SNP loci in the *PAH* gene and its upstream and downstream flanking regions (1 Mb each) were screened. Then, the DNA libraries of several family samples were mixed according to a certain ratio, hybridized, captured and sequenced on the same chip with whole-exome sequencing (WES) and whole- genome sequencing (WGS) samples using the NovaSeq 6000 to achieve efficient utilization of the chip. In terms of the cost, the final total cost of using NIPD for one family was about $700, excluding previous genetic diagnosis of the proband and parents. Regarding the used time, the whole program could be completed within 4 days, including 0.5 days for DNA extraction and library construction, 1.5 days for library hybridization and capturing, 1.5 days for sequencing and 0.5 days for data analysis. As the sequencing technique develops more, it is believed that the total cost could be further reduced and the overall process could be shortened.

Our technique has the following defects. (1) Due to the sample size limitation, there was a gray area for S_value where the fetal genotype cannot be determined. Hence, a larger sample size is needed to determine the S_value, narrow its gray area range or accurately determine its cut-off value. (2) The correlations of the sequencing depth, fetal fraction in cell free DNA and the numbers of SNP loci with fetal haplotype were not assessed. According to relevant studies, these factors and the characteristics of the target region may have a vital influence on the accuracy of NIPD [24]. Ye *et al.* investigated and evaluated the associations of fetal fraction in cell free DNA and sequencing depth with fetal haplotype accuracy. They reported that that if fetal fraction in cell free DNA is set to 10%, then to achieve an accuracy of 99% for fetal genetic maternal haplotypes inferred from 20 SNP loci, the plasma sequencing depth must reach 100 X, while only 10 SNP loci will be needed if the sequencing depth reaches 200 X [19]. Their work also simulated the relationship between fetal fraction in cell free DNA and fetal genetic maternal SNPs, and the results revealed that when fetal fraction in cell free DNA is 8%, 9% and 10%, the number of fetal genetic maternal SNP loci needed for the construction of fetal haplotypes is 20, 12 and 10, respectively, with an accuracy of 99% [19]. Although our study cannot clarify the minimum fetal fraction in cell free DNA that can determine the fetal genotype, but when the plasma sequencing depth reach 3,000 X and 270 SNP loci, and fetal fraction in cell free DNA is 3.6%, F20 were diagnosed as PKU patient. Therefore, the fetal fraction in cell free DNA reaches about 3.5%, which can be used for NIPD of PKU. (3) Another limitation of this technique is the need for a blood sample from a proband, which is not available if the proband died, so a technique for directly determining the parental haplotypes without the need for requiring the proband is very necessary in clinical practice. In future studies, we plan to develop NIPD for PKU that does not require the proband and apply it in clinical practice.

## Conclusions

In conclusion, this study shows that non-invasive prenatal testing of haplotypes based on paired-end molecular tags and weighting algorithm applied to the genotyping of fetuses with a high risk for PKU achieves an accurate detection at low fetal fraction in cell free DNA and at the early stage of pregnancy. The presented technique can serve as a first-tier screening technique for monogenic inherited diseases such as the PKU.

## Supplementary Information

**Additional file 1.** **Table S1.** Statistics of target region sequencing data. **Table S2.** Statistics of the paternal or maternal haplotypes ofthe fetuses.

## Data Availability

The raw DNA datasets (*PAH* gene sequences) used during the current study are deposited in NCBI database (Gene ID: 5053), https://www.ncbi.nlm.nih.gov/gene/?term=5053. The datasets supporting the conclusions of this article are included within the article and its additional files.

## Abbreviations

PKU: phenylketonuria
NIPD: non-invasive prenatal diagnosis
PAH: phenylalanine hydroxylase
cf-DNAs: cell-free deoxyribonucleic acids
NIPT: non-invasive prenatal test
SNP: single nucleotide polymorphism
RNA: ribonucleic acid
MLPA: multiplex ligation-dependent probe amplification
cSMART: circulating single-molecule amplification and resequencing technology
SPRT: sequential probability ratio test
RHDO: relative haplotype dosage
WES: whole-exome sequencing
WGS: whole-genome sequencing
