# Supplementary material for: Clinical application of non-invasive prenatal diagnosis of phenylketonuria based on haplotypes via paired-end molecular tags and weighting algorithm
Source: BMC Med Genomics. 2021 Dec 17;14:294. doi: 10.1186/s12920-021-01141-4 (PMC8684071; doi:10.1186/s12920-021-01141-4)
Supplement: Supplementary file 1 — Additional file 1. Table S1. Statistics of target region sequencing data. Table S2. Statistics of the paternal or maternal haplotypes ofthe fetuses. [file 12920_2021_1141_MOESM1_ESM.doc]

**Table S1. Statistics of target region sequencing data**

| **Pedigree** | **project number** | **Sample ID** | **Depth ≥20X** | **Sequencing data(Mb)** | **Capture efficiency** | **duplication rate(%)** | **sequencing depth** |
| --- | --- | --- | --- | --- | --- | --- | --- |
| F01 | HY20026816 | Proband | 99.66% | 2536 | 43.12% | 0.388 | 2000.03 |
|  | HY20026817 | Father | 100% | 3023 | 46.84% | 0.406 | 2584.41 |
|  | HY20026818 | Mother | 99.75% | 1694 | 42.91% | 0.479 | 1325.23 |
|  | HY20026819 | Fetal | 99.86% | 3074 | 35.56% | 0.616 | 2089 |
| F02 | HY20026823 | Proband | 99.75% | 2692 | 39.97% | 0.429 | 1982.9 |
|  | HY20026820 | Father | 99.99% | 2216 | 38.18% | 0.391 | 1597.08 |
|  | HY20026821 | Mother | 99.75% | 2063 | 42.43% | 0.503 | 1587.01 |
|  | HY20026822 | Fetal | 99.72% | 2933 | 40.45% | 0.616 | 2266.92 |
| F03 | HY20026827 | Proband | 99.75% | 2450 | 42.32% | 0.392 | 1907.94 |
|  | HY20026824 | Father | 100% | 2349 | 43.33% | 0.402 | 1870.72 |
|  | HY20026825 | Mother | 99.75% | 2069 | 44.12% | 0.492 | 1655.75 |
|  | HY20026826 | Fetal | 100% | 3286 | 40.73% | 0.64 | 2534.73 |
| F04 | HY201214158 | Proband | 99.91% | 1022 | 49.04% | 0.281 | 903.34 |
|  | HY201214159 | Father | 100% | 1243 | 45.15% | 0.299 | 1005.59 |
|  | HY201214160 | Mother | 99.58% | 1171 | 46.06% | 0.47 | 979.54 |
|  | HY201214161 | Fetal | 99.99% | 2711 | 47.47% | 0.513 | 2455.96 |
| F05 | HY201214162 | Proband | 99.94% | 1106 | 44.80% | 0.326 | 896.96 |
|  | HY201214163 | Father | 99.82% | 1107 | 47.67% | 0.284 | 957.72 |
|  | HY201214164 | Mother | 99.69% | 1724 | 48.87% | 0.382 | 1524.46 |
|  | HY201214165 | Fetal | 100% | 3216 | 46.96% | 0.525 | 2860.49 |
| F06 | HY201214166 | Proband | 99.74% | 1333 | 43.78% | 0.337 | 1055.82 |
|  | HY201214167 | Father | 99.94% | 1055 | 46.52% | 0.289 | 888.05 |
|  | HY201214168 | Mother | 99.70% | 1964 | 49.56% | 0.399 | 1761.54 |
|  | HY201214169 | Fetal | 100% | 3216 | 46.96% | 0.525 | 2860.49 |
| F07 | HY20120519 | Proband | 99.99% | 3326 | 37.39% | 0.222 | 2244.34 |
|  | HY20120520 | Father | 99.70% | 2726 | 36.70% | 0.17 | 1795.76 |
|  | HY20120521 | Mother | 99.99% | 3801 | 37.17% | 0.209 | 2539.28 |
|  | HY20120522 | Fetal | 100% | 5947 | 37.95% | 0.472 | 4276.11 |
| F08 | HY20110420 | Proband | 99.41% | 4093 | 24.16% | 0.372 | 1688.45 |
|  | HY20110422 | Father | 99.46% | 3200 | 30.66% | 0.357 | 1676.09 |
|  | HY20110421 | Mother | 99.19% | 3968 | 27.14% | 0.381 | 1833.86 |
|  | HY20110423 | Fetal | 99.64% | 5729 | 20.11% | 0.434 | 2058.73 |
| F09 | HY21010732 | Proband | 99.99% | 2283 | 24.22% | 0.113 | 1583.63 |
|  | HY21010733 | Father | 99.74% | 1774 | 25.05% | 0.111 | 1280.33 |
|  | HY21010738 | Mother | 100% | 4606 | 25.01% | 0.105 | 2131.72 |
|  | HY21010739 | Fetal | 99.75% | 1876 | 24.54% | 0.27 | 1113.01 |
| F10 | HY200710286 | Proband | 99.51% | 2109 | 25.48% | 0.229 | 836.02 |
|  | HY200710283 | Father | 99.64% | 2294 | 26.35% | 0.215 | 944.39 |
|  | HY200710284 | Mother | 99.54% | 2080 | 26.85% | 0.242 | 871.95 |
|  | HY200710285 | Fetal | 99.97% | 2302 | 27.12% | 0.509 | 1563.66 |
| F11 | HY20120669 | Proband | 99.74% | 3354 | 45.74% | 0.188 | 2788.78 |
|  | HY20120670 | Father | 99.99% | 3449 | 43.99% | 0.192 | 2745.92 |
|  | HY20120671 | Mother | 99.99% | 3226 | 42.97% | 0.193 | 2514.77 |
|  | HY20120672 | Fetal | 99.99% | 3665 | 43.85% | 0.488 | 3045.01 |
| F12 | HY21010792 | Proband | 99.70% | 3434 | 42.16% | 0.283 | 2602.18 |
|  | HY21010793 | Father | 99.73% | 3813 | 42.50% | 0.299 | 2895.54 |
|  | HY21010794 | Mother | 99.99% | 3973 | 43.15% | 0.258 | 3075.97 |
|  | HY21010795 | Fetal | 99.87% | 9299 | 42.24% | 0.476 | 7473.97 |
| F13 | HY21010951 | Proband | 99.98% | 2151 | 37.08% | 0.205 | 1424.17 |
|  | HY21010952 | Father | 99.97% | 2788 | 35.97% | 0.213 | 1793.36 |
|  | HY21010953 | Mother | 99.71% | 2629 | 35.65% | 0.198 | 1673.7 |
|  | HY21010954 | Fetal | 99.97% | 2524 | 34.37% | 0.42 | 1613.29 |
| F14 | HY21020956 | Proband | 100% | 3388 | 47.38% | 0.272 | 2885.8 |
|  | HY21020957 | Father | 100% | 3220 | 47.92% | 0.275 | 2774.49 |
|  | HY21020958 | Mother | 99.75% | 3240 | 47.20% | 0.251 | 2732.01 |
|  | HY21020959 | Fetal | 100% | 5366 | 47.65% | 0.502 | 4828.26 |
| F15 | HY21020960 | Proband | 100% | 1979 | 41.96% | 0.218 | 1448.35 |
|  | HY21020961 | Father | 99.75% | 3272 | 42.83% | 0.226 | 2459.17 |
|  | HY21020962 | Mother | 100% | 2139 | 43.57% | 0.21 | 1636.44 |
|  | HY21020963 | Fetal | 99.82% | 4491 | 42.48% | 0.473 | 3582.38 |
| F16 | HY21031223 | Proband | 99.99% | 4789 | 30.65% | 0.141 | 2621.8 |
|  | HY21031224 | Father | 99.74% | 7012 | 26.98% | 0.153 | 3371.61 |
|  | HY21031225 | Mother | 100% | 5590 | 28.77% | 0.139 | 2864.51 |
|  | HY21031226 | Fetal | 99.99% | 4010 | 28.62% | 0.357 | 2118.1 |
| F17 | HY21031314 | Proband | 100% | 4773 | 40.45% | 0.208 | 3434.39 |
|  | HY21031315 | Father | 99.99% | 3637 | 40.33% | 0.174 | 2603.29 |
|  | HY21031316 | Mother | 99.65% | 3743 | 42.10% | 0.186 | 2798.52 |
|  | HY21031317 | Fetal | 99.99% | 2932 | 46.88% | 0.479 | 2587.44 |
| F18 | HY21031318 | Proband | 99.96% | 4055 | 33.30% | 0.106 | 1959.04 |
|  | HY21031319 | Father | 99.95% | 3174 | 35.06% | 0.112 | 1855.53 |
|  | HY21031320 | Mother | 99.69% | 4420 | 32.91% | 0.121 | 1720.52 |
|  | HY21031321 | Fetal | 99.71% | 3495 | 35.02% | 0.209 | 1970.6 |
| F19 | HY21010734 | Proband | 99.94% | 1921 | 35.03% | 0.1 | 1495.22 |
|  | HY21010735 | Father | 99.74% | 3081 | 33.76% | 0.109 | 1742.49 |
|  | HY21010736 | Mother | 99.72% | 1849 | 34.33% | 0.07 | 1456.56 |
|  | HY21010737 | Fetal | 99.76% | 5772 | 33.87% | 0.177 | 2448.16 |
| F20 | HY20120588 | Proband | 99.99% | 2819 | 45.15% | 0.304 | 1353.96 |
|  | HY20120589 | Father | 99.55% | 2492 | 43.72% | 0.188 | 1373.45 |
|  | HY20120590 | Mother | 99.69% | 2721 | 43.86% | 0.221 | 1710.43 |
|  | HY20120591 | Fetal | 99.99% | 3544 | 43.85% | 0.489 | 3029.06 |
| F21 | HY21041672 | Proband | 99.75% | 2723 | 41.77% | 0.154 | 1994.33 |
|  | HY21041673 | Father | 99.91% | 2016 | 44.69% | 0.149 | 1578.96 |
|  | HY21041674 | Mother | 99.75% | 2252 | 43.88% | 0.143 | 1741.42 |
|  | HY21041675 | Fetal | 100% | 8380 | 42.37% | 0.44 | 6657.06 |

**Table S2. Statistics of the paternal or maternal haplotypes of the fetuses**

| **Pedigree** | **mutation type** | **sites** | **Fetal DNA fraction** | **the ratio of maternal site** | **fetal ratio** | **Predictive value of benign haplotypes** | **Predictive value of pathogenic haplotypes** | **fetal haplotype** | **Maternal haploid results** | **paternal haploid results** | **conclusion** |
| --- | --- | --- | --- | --- | --- | --- | --- | --- | --- | --- | --- |
| F1 | s1 | 221 | 0.077 | 0.488 | 0.445 | 0.488 | 0.45 | 9.556 | 1538.2401 | 326250 | affected |
|  | s2 | 131 | 0.077 | 0.482 | 0.484 | 0.444 | 0.482 | 20.250 |  |  |  |
|  | s3 | 24 | 0.077 | 0.487 | 0.463 | 0.525 | 0.487 | 2.604 |  |  |  |
|  | s4 | 125 | 0.077 | 0.477 | 0.506 | 0.477 | 0.515 | 3.053 |  |  |  |
|  | s5 | 167 | 0.077 | 0 | 0.001 | 0.038 | 0 | 37.500 |  |  |  |
|  | s6 | 231 | 0.077 | 0 | 0.029 | 0 | 0.038 | 3.053 |  |  |  |
|  | s7 | 65 | 0.077 | 1 | 0.962 | 1 | 0.962 | 76.000 |  |  |  |
|  | s8 | 123 | 0.077 | 0.999 | 0.999 | 0.962 | 1 | 37.500 |  |  |  |
| F2 | s1 | 170 | 0.087 | 0.481 | 0.448 | 0.481 | 0.438 | 3.143 | 447.2484 | 3280 | affected |
|  | s2 | 268 | 0.087 | 0.478 | 0.459 | 0.434 | 0.478 | 1.289 |  |  |  |
|  | s3 | 133 | 0.087 | 0.485 | 0.484 | 0.528 | 0.485 | 44.500 |  |  |  |
|  | s4 | 106 | 0.087 | 0.484 | 0.515 | 0.484 | 0.527 | 2.480 |  |  |  |
|  | s5 | 107 | 0.087 | 0 | 0 | 0.043 | 0 | 10.000 |  |  |  |
|  | s6 | 319 | 0.087 | 0 | 0.029 | 0 | 0.043 | 2.000 |  |  |  |
|  | s7 | 70 | 0.087 | 1 | 0.959 | 1 | 0.957 | 16.400 |  |  |  |
|  | s8 | 52 | 0.087 | 1 | 1 | 0.957 | 1 | 10.000 |  |  |  |
| F3 | s1 | 419 | 0.038 | 0.481 | 0.469 | 0.481 | 0.462 | 1.714 | 0.0321 | 1670.625 | carrier |
|  | s2 | 161 | 0.038 | 0.488 | 0.456 | 0.469 | 0.488 | 0.406 |  |  |  |
|  | s3 | 67 | 0.038 | 0.48 | 0.493 | 0.499 | 0.48 | 0.462 |  |  |  |
|  | s4 | 230 | 0.038 | 0.476 | 0.476 | 0.476 | 0.495 | 0.100 |  |  |  |
|  | s5 | 109 | 0.038 | 0 | 0.001 | 0.019 | 0 | 18.000 |  |  |  |
|  | s6 | 142 | 0.038 | 0 | 0.015 | 0 | 0.019 | 3.750 |  |  |  |
|  | s7 | 14 | 0.038 | 0.999 | 0.982 | 1 | 0.981 | 18.000 |  |  |  |
|  | s8 | 142 | 0.038 | 1 | 0.992 | 0.981 | 1 | 1.375 |  |  |  |
| F4 | s1 | 224 | 0.097 | 0.478 | 0.471 | 0.478 | 0.429 | 0.169 | 0.0002 | 0.0003 | normal |
|  | s2 | 195 | 0.097 | 0.481 | 0.434 | 0.432 | 0.481 | 0.032 |  |  |  |
|  | s3 | 38 | 0.097 | 0.486 | 0.525 | 0.534 | 0.486 | 0.244 |  |  |  |
|  | s4 | 37 | 0.097 | 0.486 | 0.476 | 0.486 | 0.534 | 0.171 |  |  |  |
|  | s5 | 187 | 0.097 | 0 | 0.038 | 0.049 | 0 | 0.276 |  |  |  |
|  | s6 | 86 | 0.097 | 0 | 0 | 0 | 0.049 | 0.100 |  |  |  |
|  | s7 | 49 | 0.097 | 1 | 1 | 1 | 0.952 | 0.100 |  |  |  |
|  | s8 | 133 | 0.097 | 1 | 0.946 | 0.952 | 1 | 0.102 |  |  |  |
| F5 | s1 | 126 | 0.05 | 0.485 | 0.468 | 0.485 | 0.46 | 2.125 | 0.018 | 7176 | carrier |
|  | s2 | 105 | 0.05 | 0.492 | 0.452 | 0.467 | 0.492 | 0.375 |  |  |  |
|  | s3 | 88 | 0.05 | 0.474 | 0.501 | 0.499 | 0.474 | 0.074 |  |  |  |
|  | s4 | 10 | 0.05 | 0.504 | 0.493 | 0.504 | 0.529 | 0.306 |  |  |  |
|  | s5 | 140 | 0.05 | 0 | 0 | 0.025 | 0 | 10.000 |  |  |  |
|  | s6 | 124 | 0.05 | 0 | 0.023 | 0 | 0.025 | 11.500 |  |  |  |
|  | s7 | 76 | 0.05 | 0.999 | 0.974 | 1 | 0.975 | 26.000 |  |  |  |
|  | s8 | 78 | 0.05 | 1 | 0.999 | 0.975 | 1 | 24.000 |  |  |  |
| F6 | s1 | 179 | 0.081 | 0.485 | 0.467 | 0.485 | 0.445 | 0.800 | 0.0072 | 139040 | carrier |
|  | s2 | 125 | 0.081 | 0.472 | 0.434 | 0.431 | 0.472 | 0.066 |  |  |  |
|  | s3 | 43 | 0.081 | 0.493 | 0.514 | 0.533 | 0.493 | 0.929 |  |  |  |
|  | s4 | 64 | 0.081 | 0.484 | 0.477 | 0.484 | 0.524 | 0.147 |  |  |  |
|  | s5 | 158 | 0.081 | 0.001 | 0 | 0.041 | 0 | 10.000 |  |  |  |
|  | s6 | 81 | 0.081 | 0 | 0.033 | 0 | 0.041 | 4.400 |  |  |  |
|  | s7 | 25 | 0.081 | 0.999 | 0.96 | 1 | 0.96 | 80.000 |  |  |  |
|  | s8 | 131 | 0.081 | 0.999 | 0.999 | 0.96 | 1 | 39.500 |  |  |  |
| F7 | S1 | 300 | 0.052 | 0.486 | 0.464 | 0.486 | 0.463 | 26.091 | 80.097 | 7500 | affected |
|  | S2 | 106 | 0.052 | 0.489 | 0.487 | 0.463 | 0.489 | 11.124 |  |  |  |
|  | S3 | 64 | 0.052 | 0.467 | 0.497 | 0.493 | 0.467 | 0.129 |  |  |  |
|  | S4 | 89 | 0.052 | 0.472 | 0.521 | 0.472 | 0.498 | 2.143 |  |  |  |
|  | S5 | 5 | 0.052 | 0 | 0.009 | 0.026 | 0 | 1.889 |  |  |  |
|  | S6 | 296 | 0.052 | 0 | 0.024 | 0 | 0.026 | 12.000 |  |  |  |
|  | S7 | 203 | 0.052 | 1 | 0.975 | 1 | 0.974 | 25.000 |  |  |  |
|  | S8 | 134 | 0.052 | 1 | 0.999 | 0.974 | 1 | 25.000 |  |  |  |
| F8 | S1 | 199 | 0.058 | 0.484 | 0.462 | 0.484 | 0.471 | 2.439 | 493.506 | 2092.5 | affected |
|  | S2 | 83 | 0.058 | 0.5 | 0.494 | 0.471 | 0.5 | 3.821 |  |  |  |
|  | S3 | 45 | 0.058 | 0.49 | 0.487 | 0.519 | 0.49 | 11.439 |  |  |  |
|  | S4 | 53 | 0.058 | 0.484 | 0.508 | 0.484 | 0.513 | 4.630 |  |  |  |
|  | S5 | 0 | 0.058 | 0 | nan | 0.029 | 0 | nan |  |  |  |
|  | S6 | 204 | 0.058 | 0 | 0.027 | 0 | 0.029 | 13.500 |  |  |  |
|  | S7 | 152 | 0.058 | 1 | 0.969 | 1 | 0.971 | 15.500 |  |  |  |
|  | S8 | 123 | 0.058 | 1 | 1 | 0.971 | 1 | 10.000 |  |  |  |
| F9 | S1 | 24 | 0.0579 | 0.495 | 0.475 | 0.495 | 0.466 | 2.336 | 0.0853 | 100 | carrier |
|  | S2 | 5 | 0.0579 | 0.495 | 0.44 | 0.466 | 0.495 | 0.476 |  |  |  |
|  | S3 | 4 | 0.0579 | 0.495 | 0.518 | 0.524 | 0.495 | 0.274 |  |  |  |
|  | S4 | 1 | 0.0579 | 0.495 | 0.484 | 0.495 | 0.524 | 0.280 |  |  |  |
|  | S5 | 46 | 0.0579 | 0 | 0.014 | 0.029 | 0 | 1.068 |  |  |  |
|  | S6 | 13 | 0.0579 | 0 | 0.027 | 0 | 0.029 | 13.846 |  |  |  |
|  | S7 | 3 | 0.0579 | 1 | 0.983 | 1 | 0.971 | 1.423 |  |  |  |
|  | S8 | 4 | 0.0579 | 1 | 1 | 0.971 | 1 | 10.000 |  |  |  |
| F10 | S1 | 94 | 0.0605 | 0.491 | 0.489 | 0.491 | 0.461 | 0.064 | 0.033 | 0.00036 | normal |
|  | S2 | 44 | 0.0605 | 0.491 | 0.484 | 0.461 | 0.491 | 3.326 |  |  |  |
|  | S3 | 73 | 0.0605 | 0.474 | 0.502 | 0.505 | 0.474 | 0.092 |  |  |  |
|  | S4 | 58 | 0.0605 | 0.469 | 0.488 | 0.469 | 0.499 | 1.692 |  |  |  |
|  | S5 | 85 | 0.0605 | 0 | 0.027 | 0.03 | 0 | 0.120 |  |  |  |
|  | S6 | 2 | 0.0605 | 0 | 0.009 | 0 | 0.03 | 0.424 |  |  |  |
|  | S7 | 40 | 0.0605 | 1 | 0.999 | 1 | 0.97 | 0.034 |  |  |  |
|  | S8 | 24 | 0.0605 | 1 | 0.975 | 0.97 | 1 | 0.210 |  |  |  |
| F11 | S1 | 199 | 0.067 | 0.485 | 0.463 | 0.485 | 0.443 | 1.126 | 0.017 | 647.431 | carrier |
|  | S2 | 157 | 0.067 | 0.477 | 0.447 | 0.443 | 0.477 | 0.120 |  |  |  |
|  | S3 | 157 | 0.067 | 0.475 | 0.497 | 0.509 | 0.475 | 0.556 |  |  |  |
|  | S4 | 142 | 0.067 | 0.471 | 0.462 | 0.471 | 0.505 | 0.221 |  |  |  |
|  | S5 | 8 | 0.067 | 0 | 0.015 | 0.034 | 0 | 1.233 |  |  |  |
|  | S6 | 211 | 0.067 | 0 | 0.028 | 0 | 0.034 | 5.091 |  |  |  |
|  | S7 | 37 | 0.067 | 1 | 0.955 | 1 | 0.966 | 3.913 |  |  |  |
|  | S8 | 25 | 0.067 | 1 | 0.999 | 0.966 | 1 | 32.500 |  |  |  |
| F12 | S1 | 38 | 0.0435 | 0.468 | 0.43 | 0.468 | 0.446 | 2.328 | 948.897 | 0 | carrier |
|  | S2 | 68 | 0.0435 | 0.468 | 0.462 | 0.446 | 0.468 | 2.551 |  |  |  |
|  | S3 | 22 | 0.0435 | 0.468 | 0.452 | 0.49 | 0.468 | 2.349 |  |  |  |
|  | S4 | 0 | 0.0435 | 0.468 | nan | 0.468 | 0.49 | nan |  |  |  |
|  | S5 | 109 | 0.0435 | 0 | 0.016 | 0.022 | 0 | 0.359 |  |  |  |
|  | S6 | 46 | 0.0435 | 0 | 0 | 0 | 0.022 | 0.000 |  |  |  |
|  | S7 | 39 | 0.0435 | 1 | 1 | 1 | 0.978 | 0.000 |  |  |  |
|  | S8 | 29 | 0.0435 | 1 | 0.975 | 0.978 | 1 | 0.130 |  |  |  |
| F13 | S1 | 209 | 0.054 | 0.483 | 0.474 | 0.483 | 0.458 | 0.605 | 0.019 | 886.6 | carrier |
|  | S2 | 72 | 0.054 | 0.485 | 0.46 | 0.458 | 0.485 | 0.068 |  |  |  |
|  | S3 | 110 | 0.054 | 0.478 | 0.488 | 0.505 | 0.478 | 1.748 |  |  |  |
|  | S4 | 63 | 0.054 | 0.484 | 0.478 | 0.484 | 0.511 | 0.175 |  |  |  |
|  | S5 | 4 | 0.054 | 0 | 0.008 | 0.027 | 0 | 2.375 |  |  |  |
|  | S6 | 109 | 0.054 | 0 | 0.022 | 0 | 0.027 | 4.400 |  |  |  |
|  | S7 | 117 | 0.054 | 1 | 0.969 | 1 | 0.973 | 7.750 |  |  |  |
|  | S8 | 105 | 0.054 | 1 | 0.999 | 0.973 | 1 | 26.000 |  |  |  |
| F14 | S1 | 198 | 0.051 | 0.478 | 0.448 | 0.478 | 0.447 | 21.540 | 994.7 | 0.002 | carrier |
|  | S2 | 116 | 0.051 | 0.472 | 0.472 | 0.447 | 0.472 | 201.409 |  |  |  |
|  | S3 | 136 | 0.051 | 0.484 | 0.464 | 0.509 | 0.484 | 2.293 |  |  |  |
|  | S4 | 39 | 0.051 | 0.486 | 0.487 | 0.486 | 0.511 | 0.050 |  |  |  |
|  | S5 | 59 | 0.051 | 0 | 0.022 | 0.026 | 0 | 0.159 |  |  |  |
|  | S6 | 4 | 0.051 | 0 | 0.004 | 0 | 0.026 | 0.186 |  |  |  |
|  | S7 | 53 | 0.051 | 1 | 1 | 1 | 0.974 | 0.000 |  |  |  |
|  | S8 | 43 | 0.051 | 1 | 0.97 | 0.974 | 1 | 0.150 |  |  |  |
| F15 | S1 | 147 | 0.063 | 0.484 | 0.444 | 0.484 | 0.457 | 3.022 | 62.567 | 231.327 | affected |
|  | S2 | 111 | 0.063 | 0.489 | 0.47 | 0.457 | 0.489 | 0.679 |  |  |  |
|  | S3 | 133 | 0.063 | 0.463 | 0.466 | 0.494 | 0.463 | 8.497 |  |  |  |
|  | S4 | 57 | 0.063 | 0.455 | 0.499 | 0.455 | 0.487 | 3.590 |  |  |  |
|  | S5 | 11 | 0.063 | 0 | 0.025 | 0.032 | 0 | 0.260 |  |  |  |
|  | S6 | 62 | 0.063 | 0 | 0.029 | 0 | 0.032 | 11.600 |  |  |  |
|  | S7 | 58 | 0.063 | 1 | 0.961 | 1 | 0.968 | 5.200 |  |  |  |
|  | S8 | 37 | 0.063 | 1 | 0.998 | 0.968 | 1 | 14.750 |  |  |  |
| F16 | S1 | 315 | 0.079 | 0.473 | 0.461 | 0.473 | 0.451 | 1.098 | 0.014 | 0.003 | normal |
|  | S2 | 97 | 0.079 | 0.49 | 0.43 | 0.451 | 0.49 | 0.342 |  |  |  |
|  | S3 | 68 | 0.079 | 0.472 | 0.511 | 0.511 | 0.472 | 0.004 |  |  |  |
|  | S4 | 114 | 0.079 | 0.488 | 0.466 | 0.488 | 0.528 | 0.363 |  |  |  |
|  | S5 | 170 | 0.079 | 0 | 0.037 | 0.04 | 0 | 0.068 |  |  |  |
|  | S6 | 1 | 0.079 | 0 | 0.003 | 0 | 0.04 | 0.082 |  |  |  |
|  | S7 | 70 | 0.079 | 1 | 1 | 1 | 0.96 | 0.000 |  |  |  |
|  | S8 | 67 | 0.079 | 1 | 0.947 | 0.96 | 1 | 0.255 |  |  |  |
| F17 | S1 | 131 | 0.09 | 0.479 | 0.423 | 0.479 | 0.44 | 3.330 | 887.071 | 0.008 | carrier |
|  | S2 | 85 | 0.09 | 0.485 | 0.481 | 0.44 | 0.485 | 10.633 |  |  |  |
|  | S3 | 54 | 0.09 | 0.472 | 0.457 | 0.517 | 0.472 | 4.014 |  |  |  |
|  | S4 | 146 | 0.09 | 0.476 | 0.53 | 0.476 | 0.521 | 6.241 |  |  |  |
|  | S5 | 112 | 0.09 | 0 | 0.059 | 0.045 | 0 | 0.237 |  |  |  |
|  | S6 | 3 | 0.09 | 0 | 0.004 | 0 | 0.045 | 0.098 |  |  |  |
|  | S7 | 46 | 0.09 | 1 | 1 | 1 | 0.955 | 0.000 |  |  |  |
|  | S8 | 33 | 0.09 | 1 | 0.93 | 0.955 | 1 | 0.357 |  |  |  |
| F18 | S1 | 138 | 0.051 | 0.496 | 0.468 | 0.496 | 0.465 | 8.519 | 292.688 | 0.03 | carrier |
|  | S2 | 19 | 0.051 | 0.49 | 0.494 | 0.465 | 0.49 | 7.795 |  |  |  |
|  | S3 | 11 | 0.051 | 0.476 | 0.472 | 0.502 | 0.476 | 7.190 |  |  |  |
|  | S4 | 19 | 0.051 | 0.525 | 0.485 | 0.525 | 0.551 | 0.613 |  |  |  |
|  | S5 | 22 | 0.051 | 0 | 0.02 | 0.026 | 0 | 0.300 |  |  |  |
|  | S6 | 6 | 0.051 | 0 | 0 | 0 | 0.026 | 0.100 |  |  |  |
|  | S7 | 0 | 0.051 | 1 | nan | 1 | 0.974 | nan |  |  |  |
|  | S8 | 0 | 0.051 | 1 | nan | 0.974 | 1 | nan |  |  |  |
| F19 | S1 | 94 | 0.063 | 0.494 | 0.497 | 0.494 | 0.468 | 0.112 | 0.032 | 0.023 | normal |
|  | S2 | 131 | 0.063 | 0.5 | 0.433 | 0.468 | 0.5 | 0.529 |  |  |  |
|  | S3 | 117 | 0.063 | 0.484 | 0.563 | 0.515 | 0.484 | 0.603 |  |  |  |
|  | S4 | 112 | 0.063 | 0.484 | 0.499 | 0.484 | 0.516 | 0.898 |  |  |  |
|  | S5 | 144 | 0.063 | 0 | 0.056 | 0.032 | 0 | 0.438 |  |  |  |
|  | S6 | 0 | 0.063 | 0 | nan | 0 | 0.032 | nan |  |  |  |
|  | S7 | 21 | 0.063 | 1 | 0.998 | 1 | 0.968 | 0.068 |  |  |  |
|  | S8 | 28 | 0.063 | 1 | 0.934 | 0.968 | 1 | 0.523 |  |  |  |
| F20 | S1 | 109 | 0.036 | 0.505 | 0.439 | 0.505 | 0.484 | 1.450 | 18.869 | 23.8 | affected |
|  | S2 | 17 | 0.036 | 0.502 | 0.508 | 0.484 | 0.502 | 4.098 |  |  |  |
|  | S3 | 9 | 0.036 | 0.484 | 0.467 | 0.502 | 0.484 | 2.058 |  |  |  |
|  | S4 | 52 | 0.036 | 0.491 | 0.542 | 0.491 | 0.509 | 1.543 |  |  |  |
|  | S5 | 4 | 0.036 | 0 | 0.021 | 0.018 | 0 | 0.143 |  |  |  |
|  | S6 | 22 | 0.036 | 0 | 0.063 | 0 | 0.018 | 1.400 |  |  |  |
|  | S7 | 2 | 0.036 | 1 | 0.975 | 1 | 0.982 | 3.571 |  |  |  |
|  | S8 | 55 | 0.036 | 1 | 0.999 | 0.982 | 1 | 17.000 |  |  |  |
| F21 | S1 | 53 | 0.0758 | 0.484 | 0.438 | 0.484 | 0.446 | 3.569 | 149.341 | 0.001 | carrier |
|  | S2 | 30 | 0.0758 | 0.484 | 0.451 | 0.446 | 0.484 | 0.149 |  |  |  |
|  | S3 | 21 | 0.0758 | 0.484 | 0.473 | 0.522 | 0.484 | 4.446 |  |  |  |
|  | S4 | 3 | 0.0758 | 0.484 | 0.477 | 0.484 | 0.522 | 0.156 |  |  |  |
|  | S5 | 12 | 0.0758 | 0 | 0.009 | 0.038 | 0 | 3.211 |  |  |  |
|  | S6 | 76 | 0.0758 | 0 | 0 | 0 | 0.038 | 0.000 |  |  |  |
|  | S7 | 78 | 0.0758 | 1 | 0.999 | 1 | 0.962 | 0.027 |  |  |  |
|  | S8 | 7 | 0.0758 | 1 | 0.95 | 0.962 | 1 | 0.242 |  |  |  |

Note: nan, not a number
